# Supplementary figures and images for: A dual computational and experimental strategy to enhance TSLP antibody affinity for improved asthma treatment
Source: PLoS Comput Biol. 2024 Mar 27;20(3):e1011984. doi: 10.1371/journal.pcbi.1011984 (PMC10971747; doi:10.1371/journal.pcbi.1011984)

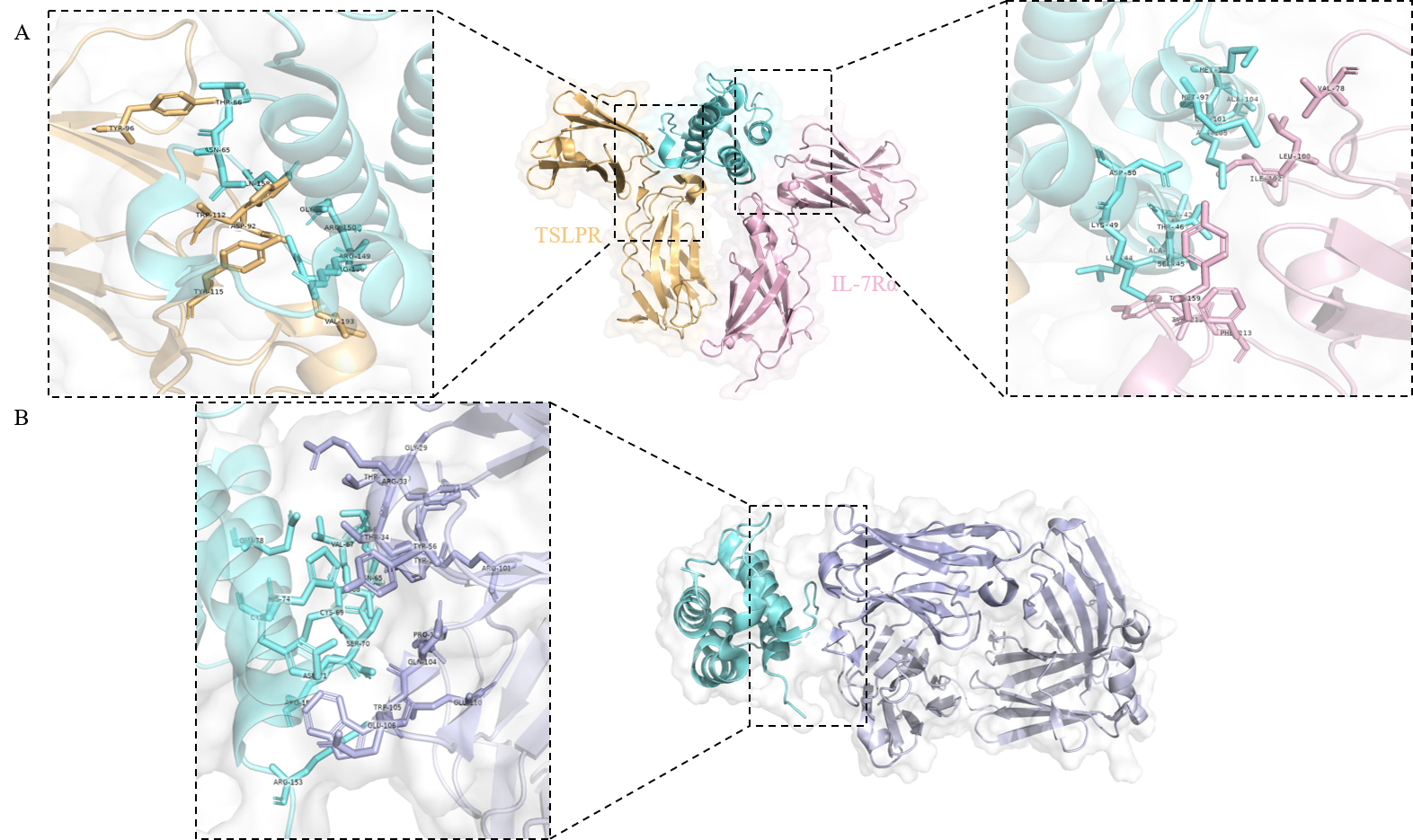

Supplement: S1 Fig — (A) Analysis of the interaction between TSLP and the TSLPR/IL-7Rα complex (PDB ID: 5J11). (B) Analysis of the interaction between AMG157 and TSLP (PDB ID: 5J13). (TIF) [file pcbi.1011984.s001.tif]
