# Supplementary material for: A dual computational and experimental strategy to enhance TSLP antibody affinity for improved asthma treatment
Source: PLoS Comput Biol. 2024 Mar 27;20(3):e1011984. doi: 10.1371/journal.pcbi.1011984 (PMC10971747; doi:10.1371/journal.pcbi.1011984)
Supplement: Attachment — Submitted filename: Review_response_PCOMPBIOL-D-23-01949.docx [file pcbi.1011984.s011.docx]

Manuscript title: A Dual Computational and Experimental Strategy to Enhance TSLP Antibody Affinity for Improved Asthma Treatment

Manuscript Number: PCOMPBIOL-D-23-01949

Authors: Yitong Lv, He Gong, Xuechao Liu, Jia Hao, Lei Xu, Zhiwei Sun, Changyuan Yu, Lida Xu

E-mail: lida.xu@hotgen.com.cn

Dear Reviewer,

Firstly, we would like to express our sincere gratitude for your careful review of our manuscript titled " A Dual Computational and Experimental Strategy to Enhance TSLP Antibody Affinity for Improved Asthma Treatment" and for providing valuable feedback. We truly appreciate your support and suggestions regarding our research.

Next, we would like to address each of your comments and provide corresponding revisions and explanations.

**Reviewer #1:**

**1. It mentioned that computer-guided homology modeling was used to generate the 3D structure of the T6 antibody variable domain. Which homology modeling method is specifically used? Furthermore, how to ensure/measure the reliability of the predicted structure? This is an important matter because the following process is based on predicted structures. Advanced protein complex structure prediction methods, such as AlphaFold-Multimer and DMFold, provide confidence in the prediction model and may help illustrate the reliability of the prediction model.**

Answer: The accuracy of the model is indeed crucial. We used Modeller for homology modeling, and the template used had a homology greater than 80% (PDB ID: 6B0W), meeting the requirements for homology modeling. Since the antibody framework region sequences are relatively conserved, the key to antibody modeling lies in modeling the CDR regions. As we cannot determine the correctness of the model, we performed alanine scanning in subsequent experiments to help confirm the accuracy of the modeled structure and the docked complex structure. AlphaFold-Multimer and DMFold do exhibit a certain level of accuracy in modeling and will greatly assist our future modeling work. We have provided additional information and suggestions regarding modeling in the Discussion section to address this aspect.

**2. Both computational tools (mCSM-PPI2 and FoldX) and experimental methods were used to perform alanine scanning. It would be better if the authors could provide performance of alanine scanning using only computational or experimental methods to demonstrate the necessity of combining the two methods. By the way, how did the authors perform the experimental alanine scan using the predicted T6 antibody structure?**

Answer: The experimental and computational alanine scanning techniques work in synergy. By assessing whether the significantly altered sites identified through experimental alanine scanning are located at the binding interface of the antigen-antibody docking complex model, we can validate the accuracy of the constructed complex models. However, since experimental alanine scanning only covers a limited number of key residues, computational software is utilized to perform alanine scanning, expanding the scope to include critical amino acids. This assists in identifying optimal mutation strategies to the greatest possible extent.

In summary, experimental alanine scanning is conducted to assist in determining the accuracy of the generated docking models, while computational alanine scanning is employed to identify critical amino acid residues for subsequent single-point mutations.

**3. It mentioned: "Consequently, these mutation points were combined, leading to the discovery that the H62Y-L49Y95D mutated strain exhibited the optimal blocking efficacy." How do you combine these mutation points? H62Y-L49Y95D represents three mutation points. L49Y comes from the first round mutation, and 95D comes from the second round mutation. What about H62Y? It seems that it should also come from the second round of mutation, but each round of mutation only mutates one amino acid at a time, which means that L95D and H62Y belong to two different variants. So how do you combine two mutations in one variant?**

Answer: We performed single-point mutations in each round of experiments. In the first round, we identified the L49Y mutation. In the second round, we conducted single-point mutations based on the L49Y mutant strain. During the second round, we discovered the mutations L95D, H62Y, H62F, L95Q, and L95Y, all of which showed improved blocking efficacy. We hypothesized that combining these mutation sites might result in even better blocking efficacy. Thus, in the third round, we performed pairwise combinations of the mutation sites identified in the second round. Eventually, we identified the H62Y-L49Y95D mutant strain.

We apologize for not providing detailed explanations of the third round of combinations in the "Second round docking and site-directed mutagenesis" section, which caused the misunderstanding. I have now added the necessary information to clarify this in the Results section.

**4. The authors state: "It was observed that the mutation of leucine to aspartate at position 95 (L95D) in the light chain exhibited a higher blocking efficacy than AMG157, ...". But Figure 5C shows that the AMG157 curve is lower than the L49Y95D curve in most areas. For context, the lower the curve, the greater the blocking efficacy.**

Answer: Thank you for pointing out this issue. The evaluation of blocking efficacy is based on calculating the IC50 values, rather than solely relying on the positioning of the curves. The IC50 value is calculated by identifying the upper and lower limits of the curve and determining the concentration value corresponding to half of the curve's range. In simple terms, a curve that is shifted to the left indicates a potentially stronger blocking ability and higher affinity for TSLP. However, specific comparisons still rely on the IC50 value. It is possible that the curves of L49Y95D and AMG157 are close to each other, leading to the misunderstanding. Therefore, apart from observing the curve graphs, it is important to consider the corresponding IC50 values. The IC50 value of L49Y95D is 0.4777, which is lower than the IC50 value of AMG157 (1.54), indicating that L49Y95D exhibits superior blocking efficacy compared to AMG157. We have provided the IC50 values of each mutated strain in the supplementary materials S5, S8, and S9 to further elucidate the results.

**5. The IC50 value mentioned on page 10, line 167, seems to be an important measure of affinity and should be briefly described. IC50 values for each variant of T6 and AFM1557 should be provided along with luciferase expression.**

Answer: Thank you for your suggestion. We did not provide the IC50 values at that time because we believed that the changes in the curves were visually evident from the graphs. However, as you pointed out, not providing specific values was incomplete. Therefore, we have added the IC50 values obtained from the experiments to supplement the information in the Results section.

**6. How to measure the affinity of antibody against TSLP? The authors provide blocking efficacy and IC50 but not explain how they relate to affinity.**

Answer: We apologize for not explaining clearly the relationship between the antibody-TSLP binding affinity and the blocking efficacy of the antibody. As mentioned in the Introduction, TSLP forms a complex with downstream TSLPR and IL-7Rα, activating the STAT5 pathway. The antibody's role is to bind to TSLP and block its activation of downstream pathways. Therefore, a higher blocking efficacy suggests that the antibody has a stronger ability to bind to TSLP and prevent its interaction with its receptor. This indicates that the antibody has a higher affinity for TSLP.

IC50 (half maximal inhibitory concentration) refers to the concentration of the measured antagonist that produces half of the maximum inhibition. In our antibody blocking experiments, we measure the fluorescent signal values. When testing the same antibody at different concentrations, a signal curve is generated (as shown in Figure 3 and Figure 5). This signal curve corresponds to an IC50 value. A smaller IC50 value indicates a stronger blocking ability of the antibody, which means a higher affinity between the antibody and TSLP.

We have already provided additional information in the Results section, specifically in the section on 'Single-Point mutations of T6 antibody'.

**7. What is the meaning of OD in Figure 1A? How does it relate to binding epitopes?**

Answer: We apologize for the lack of clarity in our previous description of the experimental results. In our experiment, we used an ELISA assay to measure the optical density (OD) as a signal value. Here is a clearer explanation of the experimental process: We immobilized AMG157 antibody on the plate, followed by the addition of a fixed concentration of TSLP (10ng/mL) and different concentrations of antibodies (AMG157 and T6). We measured the signal of the complex formed between AMG157 and TSLP on the plate. When the concentration of free AMG157 is high, it can bind to free TSLP, resulting in a decreased amount of AMG157-TSLP complex on the plate and thus a lower signal. In this scenario, if the same concentration of T6 antibody is added, the signal value decreases even further. This indicates that free AMG157 saturates and binds to TSLP, occupying the binding sites of TSLP. However, after adding T6 antibody, the signal value decreased, suggesting that in addition to the binding of TSLP and AMG157, T6 also bound to TSLP. Therefore, we can infer that the binding epitopes of T6 antibody and AMG157 antibody on TSLP are not the same. We apologize for the lack of clarity in expressing this in the results section and have now provided additional clarification in that section.

**8. The S5 table on line 216 on page 13 should be an S6 table.**

Answer: Thank you for pointing out this error. We apologize for the mistake in the table numbering. We have now corrected the error in the main text. The table on line 216 on page 13 should indeed be referred to as the S6 table.

**Reviewer #2:**

**1. This is a well written manuscript that provides important new insights. However, I was wondering why the authors opted to use transfected CHO to assess the functionality of their antibody and not a cell type that naturally expresses TSLP/R? This would provide much stronger translational value.**

Answer: Thank you for your suggestion. We chose CHO cells for several reasons. Firstly, CHO cells have high protein expression capability, which allows us to assess the functionality of the antibodies effectively. Secondly, considering the mechanism of TSLP action mentioned in the first paragraph of the Introduction, TSLP forms a complex with downstream TSLPR and IL-7Rα, activating the STAT5 pathway. Therefore, during the transfection step, we transfected TSLPR, IL-7Rα, and STAT5 into the cells at a ratio of 1:1:8 to evaluate the blocking effect of the antibodies on this pathway and assess the affinity between the antibodies and TSLP. Using a cell type that naturally expresses TSLP/R would introduce additional variables, as we would not be able to control the ratio of transfected STAT5 in those cells. Furthermore, considering other relevant literature on TSLP antibodies, they also used cell types that do not naturally express TSLP/R. (Doi: 10.1124/jpet.121.000686, Patent: CN 113069543 A)

It is possible that we did not clearly describe the experimental steps in the "Validation of Cellular Blockade Ability" section of the Methods. We apologize for the confusion, and we have already made the necessary edits to provide more detailed information in the "Validation of Cellular Blockade Ability" section of the Methods.

Thank you once again for your diligent review and constructive feedback. We believe that the revisions we have made address your concerns and significantly contribute to the quality of our work. We hope that you find this revised version suitable for publication in PLOS Computational Biology.

Thank you for your consideration.

Best regards,

Changyuan Yu
